# Determinants of Neural Tube Defects among Newborns in Amhara Region, Ethiopia: A Case-Control Study

**DOI:** 10.1155/2020/5635267

**Published:** 2020-10-30

**Authors:** Abay Woday Tadesse, Ayesheshim Muluneh Kassa, Setognal Birara Aychiluhm

**Affiliations:** ^1^Samara University, College of Medical and Health Sciences, Department of Public Health, Samara, Afar Region, Ethiopia; ^2^Dessie College of Health Sciences, Department of Nursing, Dessie, Amhara Region, Ethiopia

## Abstract

**Background:**

Worldwide, an estimated 300,000 neonates are born with neural tube defects (NTDs) each year. However, NTDs are underreported in Ethiopia though it causes substantial mortality, morbidity, disability, and psychological and economic cost in the country. Moreover, the factors attributed to NTDs were not addressed. Hence, this study intended to identify the determinants of neural tube defects in Amhara Region, Ethiopia.

**Methods:**

A case-control study design was conducted among 400 newborns (133 cases and 267 controls) who were born at randomly selected public hospitals. Cases were identified using the physician diagnosis of confirmed NTDs, and the two consecutive controls were selected using a simple random sampling technique. The data analysis was done using Stata 14.0. Variables with *p* value < 0.25 in the bivariate analysis were entered into the multivariable logistic regression model, and a corresponding 95% confidence interval was used to identify the predictors of NTDs.

**Results:**

In this study, fifty percent (48%) of the cases were contributed by anencephaly. After controlling the covariates, living in rural areas (AOR = 1.78: 95% CI 1.02, 3.11), being illiterate (AOR = 1.81: 95% CI 1.07, 4.61), being female newborn (AOR = 1.95: 95% CI 1.09, 3.50), having no ANC follow-up (AOR = 1.93: 95% CI 1.17, 5.04), and having a previous history of NTDs (AOR = 4.39: 95% CI 2.42, 7.96) were the risk factors for NTDs. However, being supplemented with folic acid or multivitamins before or during pregnancy (AOR = 0.37: 95% CI 0.21, 0.65), never having taken any substance during pregnancy (AOR = 0.42: 95% CI 0.21, 0.88), and being free from medical illnesses during pregnancy (AOR = 0.27: 95% CI 0.11, 0.69) were the protective factors of NTDs.

**Conclusion:**

The study revealed different factors associated with NTDs among newborns in the region. Therefore, comprehensive preventive strategies focused on identified risk factors are needed at regional and national levels.

## 1. Introduction

Birth defects can be defined as structural or functional abnormalities, including metabolic disorders, which are present from birth [1]. Globally, An estimated 303,000 newborns die in the first month of life every year due to congenital anomalies [2]. Neural tube defects (NTDs) are major birth defects of the brain and spine that occur early in pregnancy-related to improper closure of the embryonic neural tube [3, 4]. Neural tube defects (NTDs) are the second most common birth defect following congenital heart anomalies [1, 2, 5], and the commonest types of NTDs are spinal bifida, encephalocele, and anencephaly [3, 6, 7]. Globally, an estimated 300,000 neonates are born with neural tube defects each year [8], and 94% of severe congenital anomalies occur in low- and middle-income countries [2]. In addition, approximately 300,000 to 400,000 babies are born each year with NTDs that result in more than 88,000 deaths and 8.6 million DALYs [9–11]. However, data on NTDs are limited in lower-income countries, and a systematic review showed that many WHO member states (120/194) did not have any data on NTDs [1, 12]. Furthermore, the prevalence of NTD in Ethiopia varies from region to region, and its magnitude ranges between 0.61% and 40.3% in the country [13–18].

Neural tube defect are the second most serious leading cause of neonatal mortality next to prematurity and birth asphyxia worldwide [19]. Moreover, NTDs are associated with substantial mortality, morbidity, disability, and psychological and economic costs [5, 20, 21].

Studies conducted across the globe have identified different factors of NTDs. These include low socioeconomic status, maternal exposure to certain environmental factors (i.e., chemicals and pesticides), tobacco use during pregnancy, genetic factors, pregnancy in the late maternal age, poor intake of folic acid prior or during pregnancy, sex of the neonate, and no antenatal care [2, 15, 20, 22, 23].

Different programs, strategies, and policies at global, regional, and national levels have been tried in the past to address the burden of congenital abnormalities at large and neural tube defects in particular. These strategies include folic acid and/or multivitamins supplementation before or during pregnancy periods [9, 10, 22, 24–26] and improvement of maternal and child health status [8, 27–30]. However, the problem related to congenital anomalies especially the NTDs have not been declined in the needed manner in developing nations including Ethiopia [11, 15, 31, 32]. Furthermore, the determinants of NTDs among newborns in Ethiopia are not well addressed in the previous studies since the studies conducted in the country were limited to the magnitude of NTDs [16, 33]. Therefore, this study intended to investigate the determinants of NTDs among newborns.

## 2. Methods

### 2.1. Study Settings and Participants

The study was conducted from December 15, 2018, to January 01, 2019, among newborns in the four randomly selected public hospitals (namely, Debre Birhan, Dessie, Woldia, and Felege-Hiwot referral hospitals) those located in the Amhara Regional State, Ethiopia. The region has a total of seven referral hospitals, which serve an estimated more than 25 million populations in the region.

A hospital-based unmatched case-control study design was conducted to identify the determinants of neural tube defects (NTDs) among newborns. Neonates born after the age of viability (after 28 weeks of gestation) with a confirmed diagnosis of NTD during the study period were included as cases and those neonates born without any form NTDs during the study period were included as controls. However, pregnancies terminated before 28 weeks of gestation and neonates whose mothers were seriously ill during the study period were not included in this study.

### 2.2. Sample Size Determination and Sampling Techniques

To determine the sample size, various factors significantly associated with the outcome variable were considered, and the larger sample size was used for this study.

The required sample size was determined using the double population proportion formula with the assumptions of 95% CI, 80% power, case to control ratio of 1 : 2, and 5% contingency was allowed to compensate nonresponses. Moreover, the percent of cases exposed (*P*_2_) and the percent of controls exposed (*P*_1_) were 42.9% and 27.9% respectively [23]. The sample size was calculated using Epi info version 7.0 and the final sample size became 400 (133 cases and 267).

Based on the prestudy chart review, an estimated 6,480 neonates with NTDs were born in the randomly selected four public hospitals in the previous year (from December 2017 to 30 October 2018). Then, the cases were proportionally allocated into the randomly selected public hospitals (i.e., Debre Birhan = 30 cases, Dessie = 36 cases, Woldia = 15 cases, and Bahir Dar Felege − Hiwot hospital = 52 cases). The data collectors assigned to each hospital delivery ward interviewed the eligible case and the consecutive two controls until the required sample size was met.

### 2.3. Study Variables

The dependent variable was neural tube defects (NTDs) (present/absent). The presence of NTD cases comprises at least anyone of cases anencephaly or spinal bifida, or encephalocele based on the ICD-10 criteria [7] while the controls are neonates born without NTDs in the selected study hospitals.

Independent variables are as follows: (1) sociodemographic characteristics of the mother, (2) obstetric and medical conditions of the mother, (3) substance use during pregnancy, and (3) newborn-related factors.

### 2.4. Data Collection Tools and Procedures

The questionnaire used was adapted and modified from previous literature conducted in Ethiopia and other developing regions of Africa [14, 15, 23, 34]. The questionnaire consisted of sociodemographic characteristics, obstetric, medical, and behavioral questions relevant to the experiences of mothers during this index pregnancy.

The data were collected using face-to-face interviews guided by structured and pretested interviewer-administered questionnaire. Besides, the maternal and neonatal medical chart review was done. The same interviewer was used to interview both cases and the consecutive two controls. The outcome variable was attributed to newborns whose medical records indicated a physician or midwife diagnosis of neonates with NTD or free of NTDs. Mothers were interviewed in private rooms to ensure their privacy and to encourage participation.

Three days of training was provided for data collectors and supervisors. Eight trained midwives or nurses who work in the labor wards of each of the selected hospitals collected the data. An independent translator translated the English language into the local language (Amharic), then back to English the measurement tool. A pretest of the questionnaire was conducted with 5% (21 participants) of samples newborns delivered other than the selected public hospitals. The mothers of newborns were interviewed within four to six hours of delivery.

### 2.5. Data Processing and Analysis

The data were checked for completeness and were entered into the Epi data version 3.1. Then, the data were exported and analyzed using Stata version 14.0. The collected data were checked for normality and other assumptions. Model fitness was assessed using the Pearson or Hosmer-Lemeshow goodness-of-fit test. In addition, the correlation between the independent variables was checked.

Texts, frequency tables, graphs, mean, and standard deviations were applied to present the descriptive statistics.

Binary logistic regression analysis was done to evaluate the association of NTD with each predictor variable, and variables with a *p* value < 0.25 were entered into the multivariable logistic regression analysis models. Multivariable logistic regression analysis was performed to identify the independent predictors of NTD using adjusted odds ratios with its corresponding 95% confidence intervals (CI). Finally, the statistical significance level was declared at *p* value of less than 0.05.

## 3. Results

### 3.1. Sociodemographic Characteristics of Participants

In this study, a total of 127 cases and 254 controls were included with a response rate of 95.3%. The mean age of the participants was 29.82 years ± 6.52 SD (standard deviation).

A higher proportion of mothers in the case groups resided in rural areas compared to mothers in the control groups (59.1% and 36.2%, respectively). When comparing the highest completed educational level by mothers, a higher proportion of mothers of the cases did not attend any formal education (41.7% compared to the mother of the controls (11.4%) (Table 1).

### 3.2. Maternal Obstetric and Newborn-Related Conditions

In this study, a higher proportion of mothers of cases had no antenatal care follow-up (15.5%) compared to mothers of controls (5.5%). Moreover, a higher percent of mothers with cases were not supplemented with folic acid (77.9%) compared to mothers of controls (49.2%). Furthermore, mothers with cases had twice-higher exposure history of previous NTDs compared to mothers with controls (62.2% and 28.38%, respectively). Finally, a higher proportion of mothers of cases used substances (drugs, alcohol, khat, and cigarette) during pregnancy (29.1%) compared to the mothers of controls (9.1%) (Table 2).

### 3.3. Types of Neural Tube Defects

**Figure 1:**
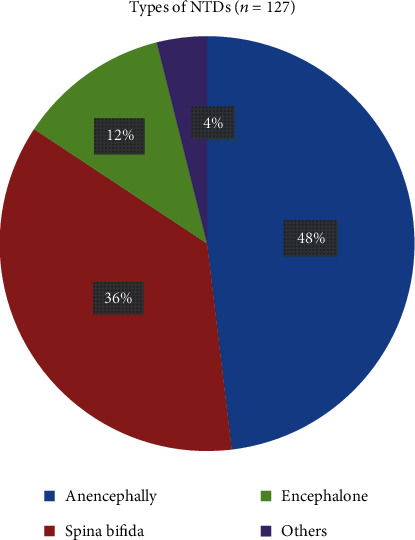
Types of NTDs among the cases of the neonates born in the hospitals, Amhara Region, Ethiopia, 2019.

In this study, the commonest type of neural tube defects among newborns is anencephaly, which accounts for 48.1% of total cases. The second and the third common types of NTDs are spinal bifida and encephalon that account for 36.2% and 11.8% of total cases, respectively. Besides, the rest 3.9% of cases were other forms of NTDs (craniorachischisis and lipomas) (Figure 1).

### 3.4. Determinants of Neural Tube Defects

The predictor variables with a *p* value of less than 0.25 in the bivariate logistic regression analysis were entered into the multivariable logistic regression analysis model to control the influence of potential confounding variables. The correlation between the independent variables was checked. Moreover, the fitness of the model was also assessed.

After controlling the covariates; women who resided in rural areas had 78% higher odds of newborns with neural tube defects (NTDs) compared to women who resided in urban residence (AOR = 1.78: 95% CI 1.02, 3.11). The likelihood of NTDs was 81% higher among newborns who had illiterate mothers compared to those who had mothers attended tertiary education levels (AOR = 1.81: 95% CI 1.07, 4.61). Female newborns had twice-higher odds of NTDs compared to male neonates (AOR = 1.95: 95% CI 1.09, 3.50). Women who had no antenatal care visits had twice-greater odds of newborns with NTDs compared to those who had antenatal care visits (AOR = 1.93: 95% CI 1.17, 5.04). The odds of NTDs were less by 63% among newborns whose mothers had taken folic acid before or during pregnancy compared to those women who were not supplemented with folic acids (AOR = 0.37: 95% CI 021, 0.65). Women who had a previous history of NTDs had 4-folds higher odds of newborns with NTDs compared to their counterparts (AOR = 4.39: 95% CI 2.42, 7.96). The likelihood of NTDs was 58% lesser among neonates whose mothers never used any substance during pregnancy compared to those who were taking substance (drugs, alcohol, khat, and cigarette) (AOR = 0.42: 95% CI 0.21, 0.88). Moreover, women who had experienced medical illnesses during pregnancy had 73% lesser odds of neonates with NTDs compared to that experienced medical illness during pregnancy (AOR = 0.27: 95% CI 0.11, 0.69). Finally, being a female neonate was the independent predictor of NTD compared to male neonates (AOR = 1.95: 95% CI 1.09, 3.50). However, having planned pregnancy, maternal age, having a history of adverse birth outcomes (i.e., abortion, PTB, LBW, and stillbirth) before this index pregnancy, and mode of delivery were not associated with neonatal NTDs in this study (Table 3).

## 4. Discussion

Defects of the neural tube involve the imperfect development of the neuropore during embryogenesis and the subsequent maldevelopment of the adjacent bone and mesenchymal structures [35]. In our study, nearly fifty percent (48%) of the NTD cases were contributed by anencephaly. This finding is similar to studies conducted in Addis Ababa Teaching Hospitals, Ethiopia (54.1%) [15], Tigray, Northern Ethiopia (66.4 per 10,000 NTD cases) [17], Gujarat hospital, India (26%) [31], and Southwest Iran (86.8%) [36]. This could be justified by the fact that anencephaly is the most common NTDs, and it developed before any of the central nervous system anomalies, developed within 1 month of conception [37]. Thus, this might be the reason for its dominancy in the current study and previous studies conducted across the world.

Studies indicated that supplementation of folic acid three months before or during pregnancy can decrease NTDs by 50-70% [24, 38, 39]. Our study revealed women who had taken folic acid before or during pregnancy had 63% less risk of births with NTDs compared to those who were not supplemented with folic acids. This finding is similar to studies conducted in Addis Ababa Teaching Hospitals, Ethiopia [15], Tigray, Northern Ethiopia [17], and Northwest Ethiopia [18]. Moreover, the result is consistent with studies conducted in Hungary [40], Adama hospital, Ethiopia [41], Jordan [42], Saudi Arabia [43], and Kingdom of Saudi Arabia [44]. Thus, in developing nations where the practice of preconception folic acid supplementation is negligible [16], folic acid fortification and supplementation policies and programs are mandatory to reduce the burden on NTDs [45–47].

In this study, women who had a previous history of NTDs had 4-folds higher odds of newborns with NTDs compared to their counterparts. This finding is very consistent with a study done in Addis Ababa City Administrative and Amhara Region, Ethiopia [14], a study conducted in central Iran [48], and a study conducted in western Iranian obstetrical centers [49]. Thus, folic acid supplementation has been shown to reduce the incidence and recurrence of NTDs [37, 39], and women with previous exposure to NTDs problem are recommended to take the maximum (4 to 5 mg) dose of folic acid on a daily basis [26, 50].

Previous studies recommended women to avoid harmful substances (i.e., tobacco, illicit drugs, and alcohol) during their pregnancy period [3, 51]. Similarly, the current study suggested that the likelihood of NTDs was 58% lesser among neonates whose mothers never used any substance during pregnancy compared to those who were taking substance (alcohol, khat, illicit drugs, and cigarette). This finding is consistent with a study done in North America [52], and a study conducted in western Iranian obstetrical centers [49].

In this study, women who resided in rural areas had 78% higher odds of newborns with neural tube defects (NTDs) compared to women who resided in urban residences. This is similar to a study conducted in Tigray regional state of Ethiopia [23]. Women who resided in rural areas that are inaccessible to the available health services might explain this. Thus, they are unable to get counseling and supplementation with folic acid that prevent the occurrence of NTDs.

Studies revealed that women with no education gave birth with NTDs compared to literate women [53]. Similarly, in the current study, the likelihood of NTDs was 81% higher among newborns who had illiterate mothers compared to those who had mothers who attended tertiary education levels. In the rural part of Ethiopia, most the women have not attended formal education, and they are not aware of the available health services in the existing health facilities. Consequently, illiterate women are more prone to have birth with NTDs than those who have attended some forms of education.

Women who had no antenatal care visits had twice-greater odds of newborns with NTDs compared to those who had antenatal care visits. This finding is similar to a study done in Northwest Ethiopia [18], and a study conducted in western Iranian obstetrical centers [49]. Women who have no ANC visits during pregnancy will not have information when to take folic acid supplementation. Thus, these women are more prone to develop many complications related to pregnancy, birth, and postpartum causes, and they will end up their pregnancy with bad outcome including NTDs compared to those have ANC follow-ups.

Women who had experienced medical illnesses during pregnancy had 73% lesser odds of neonates with NTDs compared to that experienced medical illness during pregnancy. This finding is in line with a study conducted in Northwest Ethiopia [18]. Therefore, women with any medical problems during their pregnancy and over-the-counters need to be given attention to avert the burden on congenital anomalies [3]. Thus, women with medical illness before or during pregnancy may have a low intake of foods enriched with folic acid that prone them to have birth with NTDs.

In this study, being female neonates was 2-folds a greater risk of NTD compared to male neonates. This is similar to studies conducted in Addis Ababa Teaching Hospitals, Ethiopia [15], Tigray regional state of Ethiopia [23], Batna Region, Algeria [54], Colorado, United States [53], Saudi Arabia [43], and five states of Northern China [55]. The mechanism on how the occurrence of NTDs varies between sexes is not well understood [21, 39]. Thus, there is no single justification why neural tube defect affects more females compared to males.

### 4.1. Limitations of the Study

First, the study deals with personal and sensitive behaviors, such as substance use during pregnancy. Thus, this might introduce social desirability bias. Second, the study was facility-based, in which institutional delivery is very low, and this study may not represent as close to 74% of deliveries which take place at home in the region. Lastly, the study did not address the genetic, syndromic, and chromosomal causes of NTD that are not preventable by folic acid.

## 5. Conclusion

The study identified different factors associated with NTDs among newborns in the region. After controlling the effect of covariates, residence, maternal education level, sex of the newborn, antenatal care follow-ups, previous history of birth with NTDs, folic acid intake, substance use during pregnancy, and medical illnesses during pregnancy were the independent predictors of NTDs among neonates. Therefore, comprehensive preventive strategies focused on identified risk factors are needed at regional and national government levels. Further research to address the genetic factors of NTDs is recommended.

## Figures and Tables

**Table 1:** Sociodemographic characteristics of participants who gave birth at public referral hospitals, Amhara Region, Ethiopia, 2019.

Predictor variables	Category of variables	Cases	Controls	*X* ^2^, *p* value
		*N* (%)	*N* (%)	
Age				
	18-27	40 (31.5)	96 (37.8)	3.15, *p* = 0.207
	28-34	60 (47.2)	96 (37.8)	
	≥35	27 (21.3)	62 (24.4)	

Residence				
	Rural	75 (59.1)	92 (36.2)	17.93, *p* = 0.000
	Urban	52 (40.9)	162 (63.8)	

Education level of mother				
	Illiterate	53 (41.7)	29 (11.4)	59.16, *p* = 0.000
	Primary	23 (18.1)	112 (44.1)	
	Secondary	32 (25.2)	92 (36.2)	
	Tertiary	19 (15.0)	21 (8.3)	

Marital status				
	Single	8 (6.3)	13 (5.1)	0.23, *p* = 0.634
	Married	119 (93.7)	241 (94.9)	

Sex of the newborn				
	Male	38 (29.9)	120 (47.2)	10.47, *p* = 0.001
	Female	89 (70.1)	134 (52.8)	

**Table 2:** The maternal obstetric, behavioral, and newborn-related conditions, Amhara Region, Ethiopia, 2019.

Predictor variables	Category of variables	Cases	Controls	*X* ^2^, *p* value
		*N* (%)	*N* (%)	
Parity				
	Multipara	82 (64.6)	163 (64.2)	0.015, *p* = 0.940
	Primipara	45 (35.4)	91 (35.8)	

ANC follow-up for this index pregnancy				
	Yes	108 (85.0)	240 (94.5)	9.56, *p* = 0.002
	No	19 (15.0)	14 (5.5)	

Gestational age at birth				
	<37 weeks	11 (8.7)	21 (8.3)	0.017, *p* = 0.896
	≥37 weeks	116 (91.3)	233 (91.7)	

Folic acid supplemented prior to or during pregnancy				
	No	99 (77.9)	125 (49.2)	28.87, *p* = 0.000
	Yes	28 (22.1)	129 (50.8)	

Planned pregnancy				
	Yes	51 (40.2)	93 (36.6)	0.45, *p* = 0.501
	No	76 (59.8)	161 (63.4)	

Previous history of NTDs				
	No	48 (37.8)	182 (71.7)	40.57, *p* = 0.000
	Yes	79 (62.2)	72 (28.3)	

Medical problems during this pregnancy (PROM, DM, HTN, HIV, UTI, etc.)				
	Yes	18 (14.2)	19 (7.5)	4.33, *p* = 0.038
	No	109 (85.8)	235 (92.5)	

Previous adverse birth outcomes (stillbirth, PTB, LBW, abortion, and SGA)				
	Yes	25 (19.7)	32 (12.6)	3.34, *p* = 0.068
	No	102 (80.3)	222 (87.4)	

Substance use during pregnancy (drugs, alcohol, khat, and cigarette)				
	Yes	27 (29.1)	23 (9.1)	25.73, *p* = 0.000
	No	90 (70.9)	231 (90.9)	

Onset of labor				
	Spontaneous	115 (90.6)	238 (93.7)	1.23, *p* = 0.267
	Induced	12 (9.4)	16 (6.3)	

Mode of delivery				
	SVD	105 (82.7)	224 (88.2)	2.23, *p* = 0.527
	C-section	14 (11.0)	19 (7.5)	
	Instrumental assisted delivery	8 (6.3)	11 (4.3)	

Weight of the newborn				
	<2500	87 (68.5)	9 (3.5)	189.56, *p* = 0.000
	≥2500	40 (31.5)	245 (96.5)	

Key: DM, diabetes mellitus; HTN, hypertension; HIV, human immune virus; UTI, urinary tract infection; PROM, premature rupture of membrane; PTB, preterm birth; LBW, low birth weight; SGA, small for gestational age; ANC, antenatal care; NTD, neural tube defects.

**Table 3:** Determinants of NTDs among neonates born at public hospitals, Amhara Region, Ethiopia, 2019.

Predictor variables		Birth outcomes		COR (95% CI)	AOR (95% CI)
		Cases	Controls		
Residence	Rural	75 (59.1)	92 (36.2)	2.54 (1.64, 3.93)	1.78 (1.02, 3.11)∗
	Urban	52 (40.9)	162 (63.8)	1.00	1.00

Mothers' age (in completed years)	18-27	40 (31.5)	96 (37.8)	1.00	1.00
	28-34	60 (47.2)	96 (37.8)	1.50 (0.92, 2.44)	1.74 (0.92, 3.30)
	≥35	27 (21.3)	62 (24.4)	1.04 (0.58, 1.87)	0.78 (0.36, 1.69)

Mothers' completed educational level	Illiterate	53 (41.7)	29 (11.4)	2.02 (0.93, 4.53)	1.81 (1.07, 4.61)∗
	Primary	23 (18.1)	112 (44.1)	0.23 (0.11, 0.48)	0.13 (0.05, 0.35)
	Secondary	32 (25.2)	92 (36.2)	0.38 (0.18, 0.81)	0.30 (0.12, 0.75)
	Tertiary	19 (15.0)	21 (8.3)	1.00	1.00

Sex of the newborn	Male	38 (29.9)	120 (47.2)	1.00	1.00
	Female	89 (70.1)	134 (52.8)	2.09 (1.34, 3.29)	1.95 (1.09, 3.50)∗

ANC follow-up	Yes	108 (85.0)	240 (94.5)	1.00	1.00
	No	19 (15.0)	14 (5.5)	3.02 (1.46, 6.23)	1.93 (1.17, 5.04)∗

Folic acid supplemented prior to or during pregnancy	No	99 (77.9)	125 (49.2)	1.00	1.00
	Yes	28 (22.1)	129 (50.8)	0.27 (0.17, 0.44)	0.37 (0.21, 0.65)∗

Pregnancy was planned	Yes	51 (40.2)	93 (36.6)	1.00	1.00
	No	76 (59.8)	161 (63.4)	0.86 (0.55, 1.33)	0.94 (0.54, 1.66)

Previous history of NTD(s)	No	48 (37.8)	182 (71.7)	1.00	1.00
	Yes	79 (62.2)	72 (28.3)	4.16 (2.65, 6.53)	4.39 (2.42, 7.96)∗

Substance use during pregnancy (alcohol, khat, cigarette, and drugs**)**	Yes	27 (29.1)	23 (9.1)	1.00	1.00
	No	90 (70.9)	231 (90.9)	0.24 (0.14, 0.43)	0.42 (0.21, 0.88)∗

Previous adverse birth outcomes (stillbirth, PTB, LBW, abortion, and SGA)	Yes	25 (19.7)	32 (12.6)	1.00	1.00
	No	102 (80.3)	222 (87.4)	0.59 (0.33, 1.05)	0.61 (0.28, 1.29)

Medical problems during this pregnancy (PROM, DM, HTN, HIV, UTI, etc.)	Yes	18 (14.2)	19 (7.5)	1.00	1.00
	No	109 (85.8)	235 (92.5)	0.49 (0.25, 0.96)	0.27 (0.11, 0.69)∗

Mode of delivery	SVD	105 (82.7)	224 (88.2)	1.00	1.00
	C-section	14 (11.0)	19 (7.5)	1.57 (0.76, 3.25)	2.23 (0.84, 5.91)
	Instrumental delivery	8 (6.3)	11 (4.3)	1.55 (0.61, 3.97)	2.05 (0.63, 6.59)

Key: AOR, adjusted odds ratio; COR, crude odds ratio; CI, confidence interval; ∗*p* < 0.05. DM, Diabetes Mellitus; HTN, Hypertension; HIV, Human Immune Virus; UTI, Urinary Tract Infection; PROM, Premature Rupture of Membrane; PTB, Preterm Birth; LBW, Low Birth Weight; SGA, Small for Gestational Age; ANC, Antenatal Care; NTDs, Neural Tube Defects.

## Data Availability

All materials and data related to this article are included in the main document of the manuscript. However, if anyone has interested to have raw data, he/she can contact the corresponding author.

## Abbreviations

ANC: Antenatal care
EDHS: Ethiopian Demographic and Health Survey
GA: Gestational age
NICU: Neonatal intensive care unit
NTDs: Neural tube defects
WHO: World Health Organization.
